# Interpreting Mendelian-randomization estimates of the effects of categorical exposures such as disease status and educational attainment

**DOI:** 10.1093/ije/dyab208

**Published:** 2021-09-27

**Authors:** Laurence J Howe, Matthew Tudball, George Davey Smith, Neil M Davies

**Affiliations:** Medical Research Council Integrative Epidemiology Unit, University of Bristol, Bristol, UK; Population Health Sciences, University of Bristol, Bristol, UK; Medical Research Council Integrative Epidemiology Unit, University of Bristol, Bristol, UK; Population Health Sciences, University of Bristol, Bristol, UK; Medical Research Council Integrative Epidemiology Unit, University of Bristol, Bristol, UK; Population Health Sciences, University of Bristol, Bristol, UK; Medical Research Council Integrative Epidemiology Unit, University of Bristol, Bristol, UK; Population Health Sciences, University of Bristol, Bristol, UK; K.G. Jebsen Center for Genetic Epidemiology, Department of Public Health and Nursing, NTNU, Norwegian University of Science and Technology, Trondheim, Norway

**Keywords:** Mendelian randomization, liability, educational attainment, categorical exposures

## Abstract

**Background:**

Mendelian randomization has been previously used to estimate the effects of binary and ordinal categorical exposures—e.g. Type 2 diabetes or educational attainment defined by qualification—on outcomes. Binary and categorical phenotypes can be modelled in terms of liability—an underlying latent continuous variable with liability thresholds separating individuals into categories. Genetic variants influence an individual’s categorical exposure via their effects on liability, thus Mendelian-randomization analyses with categorical exposures will capture effects of liability that act independently of exposure category.

**Methods and results:**

We discuss how groups in which the categorical exposure is invariant can be used to detect liability effects acting independently of exposure category. For example, associations between an adult educational-attainment polygenic score (PGS) and body mass index measured before the minimum school leaving age (e.g. age 10 years), cannot indicate the effects of years in full-time education on this outcome. Using UK Biobank data, we show that a higher educational-attainment PGS is strongly associated with lower smoking initiation and higher odds of glasses use at age 15 years. These associations were replicated in sibling models. An orthogonal approach using the raising of the school leaving age (ROSLA) policy change found that individuals who chose to remain in education to age 16 years before the reform likely had higher liability to educational attainment than those who were compelled to remain in education to age 16 years after the reform, and had higher income, lower pack-years of smoking, higher odds of glasses use and lower deprivation in adulthood. These results suggest that liability to educational attainment is associated with health and social outcomes independently of years in full-time education.

**Conclusions:**

Mendelian-randomization studies with non-continuous exposures should be interpreted in terms of liability, which may affect the outcome via changes in exposure category and/or independently.

Key Messages

- Genetic variants influence categorical exposures via their effects on liability, an underlying latent variable.
- Mendelian-randomization analyses with categorical exposures may be biased by effects of liability that are independent of the exposure.
- Exposure-independent liability effects can be detected using populations in which the exposure is invariant.
- Liability to educational attainment likely influences smoking behaviour and risk of glasses use independently of measured education.

## Introduction

Mendelian randomization can be implemented as an instrumental variables analysis using genetic variants to evaluate causal relationships of potential exposures (e.g. low-density lipoprotein cholesterol) on outcomes (e.g. coronary heart disease).^1^^,^^2^ For example, the Mendelian-randomization Wald estimator—the ratio of the associations of the genetic variants with the outcome and the exposure—can be used to estimate the effect of an exposure on an outcome. Mendelian-randomization analyses require the three core instrumental variable assumptions: (i) the genetic variants are associated with the exposure (relevance), (ii) there are no unmeasured confounders of the genetic variant–outcome association (independence) and (iii) that the genetic variants only influence the outcome via their effect on the exposure (the exclusion restriction).^3–5^ Mendelian randomization has been widely used to estimate the effects of continuous exposures and to estimate the effects of binary and ordinal categorical exposures such as Type 2 diabetes status^6–9^ and educational attainment.^10–12^

**Figure 1:**
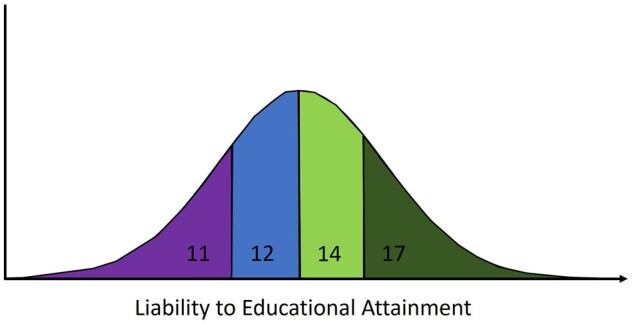
Years spent in full-time educational attainment as a function of underlying continuous liability. Individuals in the population can spend 11, 12, 14 or 17 years in full-time education (started at the age of 4 years and left aged 15, 16, 18 and 21 years, respectively). The educational-attainment category is determined by their underlying liability with respect to population-level thresholds.

Conceptually, binary exposures, such as disease status, can be modelled by assuming an underlying continuous liability—a normally distributed latent (unmeasured) variable.^13–20^ Liability models can be deterministic (liability explains all of the variation in the exposure) as in the Falconer liability-threshold model.^13^^,^^16^ In this model, an individual’s binary disease status is completely determined by whether the value of their liability to the disease is higher or lower than a threshold value. Here, liability is a combined measure of all sources of variation influencing disease risk: genetic variation, the environment and stochastic/chance variation.^13^^,^^15^^,^^21^ Although the liability-threshold model is most often used for binary disease states, it can be extended to ordinal categorical phenotypes,^22^ i.e. any phenotype with a finite number of possible values but a clear continuous ordered dimension. For example, considering years spent in full-time education, individuals across a population have an underlying liability to educational attainment and their duration in the full-time education category is determined by their liability with respect to multiple population-level thresholds (Figure  1/Box 1).

Box 1 How the interpretation of liability to educational attainment can differ across study populations

**1. Differences across countries**

In some countries (e.g. the UK), remaining in school for an additional year is an indicator of an individual choosing to remain in education for longer. However, in other countries, students may remain in school for longer if they are held back a year (e.g. the USA). Therefore, an additional year of education does not necessarily mean higher liability to education. In the USA, individuals who are kept back a year are likely to have a lower liability to educational attainment than those who are not. This caveat can be mitigated by defining years of educational attainment using qualifications rather than the years an individual spent in full-time education.

**2. Differences over time**

Schools and universities have changed greatly over the last century. In most countries, the proportion of individuals remaining in school after the age of 16 years has substantially increased, with the proportion of individuals attending university also rising. This means that the distribution of individuals attending university has changed over time to include individuals with lower liability to education than previously. As a result, the implications of an additional year of education or attending university may be very different in 1950 than in 2020.

**3. Differences in type of education**

Even within countries, education is a very heterogenous phenotype, between different types of educational establishment, and even within schools and universities. For example, there may be large differences in liability to education between individuals who attend one university or another, or between individuals who attend private schools or state-funded education.

These differences across countries, over time and across different types of institution will not necessarily be reflected in the ordinal measures of education used in many studies. Hence, it is useful to conceptualize educational measures in terms of the participant’s underlying liability to education, rather than as a highly specific effect of making a specific educational choice (e.g. choosing to remain in school at age 16 years or attending a private school or choosing to go to university).

Here, we start by discussing how to interpret the effects of categorical exposures in Mendelian-randomization analyses. In particular, we describe three possible causal mechanisms between a categorical exposure and an outcome. Next, we outline how performing analyses in a population in which the categorical exposure is invariant (all individuals have the same value for the categorical exposure) can be used to inform the most likely causal mechanism. We then use empirical data from UK Biobank to evaluate whether educational-attainment polygenic scores (PGSs), used as proxies for liability to educational attainment, are associated with childhood phenotypes [glasses wearing, smoking initiation and body mass index (BMI)]. As the outcomes are measured in children below the minimum school leaving age, associations cannot reflect effects of additional educational attainment (years in full-time education). Finally, we use a previous UK-wide school-reform and UK Biobank data to further investigate the mechanism via which educational attainment affects later-life outcomes.

## Theoretical basis for estimating the effects of categorical exposures using Mendelian randomization

The interpretation of non-continuous exposures in Mendelian-randomization analyses has been previously discussed.^23–25^ Taylor *et al.* described how Mendelian-randomization analyses of the effects of smoking using categorical exposures based on reported cigarettes per day, are likely to be biased compared with more precise measures such as blood cotinine.^23^ Burgess and Labrecque discussed how to interpret the effects of binary exposures. In particular, they note that the exclusion-restriction assumption requires that the genetic variants do not influence the outcome except via the binary exposure status.^25^ For example, genetic variants may only influence chemotherapy treatment status via cancer diagnosis. If this does not hold, e.g. if the underlying latent continuous trait influences the outcome independently of the binary exposure, then this would lead to bias in Mendelian-randomization estimates.^25^^,^^26^ We extend previous discussion by outlining theoretical models for interpreting the effects of categorical (binary or ordinal) exposures.

Generally, Mendelian-randomization studies with binary or ordinal categorical exposures are interested in estimating the effect of a change in category (C). For example, estimating the effect of being exposed or not for a binary exposure (e.g. the effect of having a disease or not) or the effect of increasing by a level of categorical variable (e.g. the effect of getting 12 rather than 11 years of education)—rather than the effects of the underlying liability to the categorical variable (L).^24^^,^^27^ However, genetic instruments, with the possible exception of some variants relating to monogenic phenotypes, are not deterministic and so influence L. For many phenotypes, L it is difficult to conceptualize in terms of an observable phenotype as it is often a latent measure of an individual’s risk of a phenotype. For orofacial clefts, L could be thought of as a phenotype relating to lip fusion *in* *utero*; for a myocardial infarction (MI), L would relate to arterial phenotypes such as atherosclerosis; and for educational attainment, *L* could be a measure of an individual’s enthusiasm for education.

In many instances, L could plausibly affect an outcome O via pathways involving changes in C and also via pathways independently of C (I) (Equations 1 and 2). For example, liability to MI could clearly impact mortality via having an MI event but underlying liability to MI relating to atherosclerotic disease could also affect mortality amongst individuals independently of having an MI event. Similarly, a combination of Type 2 diabetes and related metabolic disturbances may contribute to the increased incidence of coronary heart disease risk amongst Type 2 diabetics.^28^^,^^29^ It follows that the association between genetic instruments for a categorical exposure and O will capture effects of L on O, via both I and C. If the effects via I are non-zero, then this would lead to a violation of the exclusion-restriction assumption in a Mendelian-randomization analysis:

(1) O=kL+ ∈

(2) $$O=k_{1}C+k_{2}I\;+\;\in$$

where k represents the total effect of L on O, $k_{1}$ represents the effect of L on O mediated by C, $k_{2}$ represents the effect of L on O mediated by I and ∈ represents the remaining sources of variation in O.

Three possible causal mechanisms between a categorical exposure and an outcome are:

- the liability-exclusive model: L influences O but via pathways I, independently of C;
- the threshold-exclusive model: L influences O only via threshold effects relating to changes in C (stepwise);
- the combined model: L influences O via both I and C.

**Figure 2:**
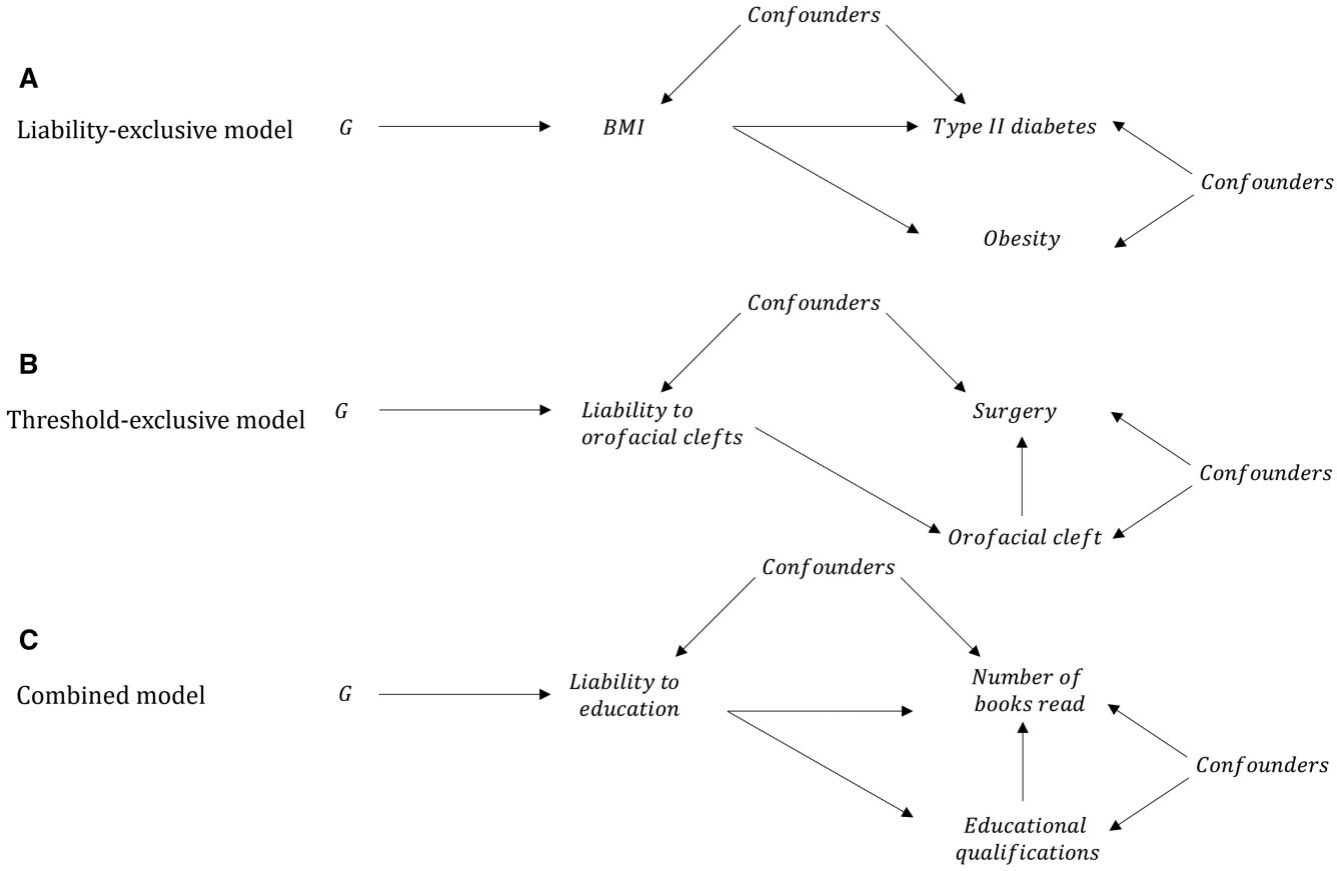
Causal graph illustrations of interpreting causal relationships between non-continuous exposures and outcomes. Illustrations of the liability-exclusive, threshold-exclusive and combined models for interpreting causal relationships between ordinal categorical exposures and outcomes. (A) Under the liability-exclusive model, liability influences the outcome solely via effects that are independent of the exposure category. For example, an MR study of obesity (i.e. BMI > 30) and Type 2 diabetes would suggest that obesity increases the risk of Type 2 diabetes. However, this effect is likely to be solely due to the effects of continuous body mass index (liability to obesity) rather than threshold effects relating to body mass index categories. (B) In the threshold-exclusive model, liability to the exposure influences the outcome entirely via threshold effects relating to the categories of the exposure (i.e. a stepwise effect). For example, individuals born with an orofacial cleft are likely to have corrective surgery but individuals who do not develop an orofacial cleft will not, irrespective of their underlying liability to orofacial clefts. (C) In the combined model, liability influences the outcome via the categorical exposure and via pathways independently of the categorical exposure. For example, spending longer in full-time education involves reading books but individuals with high liability to educational attainment may also be more likely to read books independently of educational attainment. G, genetic factors; BMI, body mass index.

Mendelian-randomization estimates can be interpreted as reflecting each of these models under different assumptions. For the estimates to reflect the liability-exclusive model [(i) above], we must assume that changing C, holding L constant, has no effect on the outcome. For example, a Mendelian-randomization study of obesity may suggest that obesity affects the risk of Type 2 diabetes. However, this relationship is likely entirely driven by the underlying variable BMI. Alternatively, we could assume that the estimates solely reflect the effect of the threshold model [(ii) above] and that L has no effect on the outcome except via C. For example, children born with orofacial clefts require surgery; this is likely to be entirely mediated by whether someone has a cleft or not, with the underlying liability having no impact independently of cleft status. Finally, it is possible that the estimates reflect a combination of the two models, i.e. effects of L mediated by, and independent of, C (Figure  2).

## Evaluating the ‘threshold-exclusive’ model

The ‘threshold-exclusive’ model can be tested using genetic variants associated with the exposure in a population in which there is no variation in the categorical exposure. Consider the example of genetic-risk variants associated with coronary heart disease in adults amongst children—a group likely to be free of coronary heart disease.^30^ In this context, assuming that the variants identified in adults influence the liability to coronary heart disease in childhood (relevance), no confounders of the genetic instrument–outcome association (independence) and that changes to the genetic components of liability lead to the same changes in the outcome as changes to environmental components (gene–environmental equivalence),^31^ associations between a coronary heart disease genetic score and outcomes (e.g. metabolites in childhood^28^^,^^30^) would be indicative of effects of liability to coronary heart disease independently of disease status. Liability effects in this context could be interpreted as evidence that the outcome could be a phenotype on the pathway to coronary heart disease. For example, metabolic disturbance in childhood could be an early precursor of atherosclerosis. We note an additional caveat that causal relationships may differ between childhood and adulthood^32^ and so this approach is sensitive to heterogeneity in effects by age. For example, liability to coronary heart disease could influence metabolites in adults but not children.

**Figure 3:**
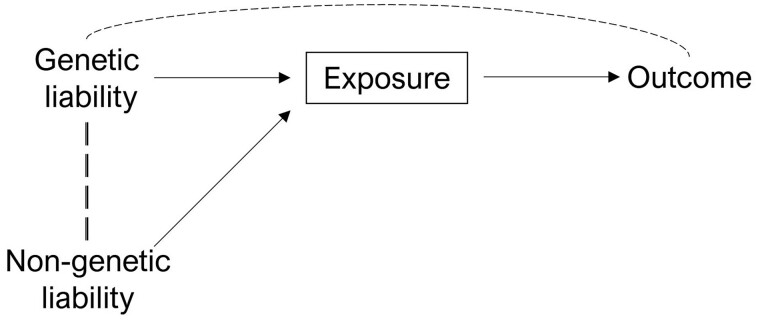
A causal graph illustrating the potential for associations between genetic and non-genetic liability when conditioning on exposure category. By stratifying on the exposure category, associations could be induced between genetic and non-genetic determinants of liability. For example, if a diseased case has low genetic liability to a disease then, depending on the model, they may be more likely to have higher non-genetic liability (environment/stochastic). Dotted lines illustrate induced correlations and a backdoor path between genetic liability and the outcome that could be induced by conditioning on the exposure if there are confounders of the exposure–outcome relationship.

Another approach could be to stratify individuals by exposure category, i.e. split a study of elderly adults into individuals with and without a disease and determine whether the genetic score is associated with the outcome within each stratum. This approach is not generally advisable because stratifying on the exposure will likely induce collider bias. For example, disease-free individuals with high genetic risk for the disease are likely to have low non-genetic liability to the disease, e.g. coronary heart disease^33^ (Figure  3). Similarly, measurement error could induce associations in the stratified analysis even if effects are entirely via threshold effects.

Analysis in stratified subgroups can be susceptible to collider bias but stratification on factors that cannot be influenced by the exposure and the outcome is unlikely to induce bias (e.g. sex or age).^34^^,^^35^ If an exposure is invariant in either sex, in an age group or perhaps in a country, it is possible to evaluate the ‘threshold-exclusive’ model without collider concerns. For example, a similar approach has been used in previous Mendelian-randomization studies of alcohol consumption in East Asian populations to investigate whether associations between alcohol-related genetic variants and outcomes are via alcohol consumption. In these populations, women have very low alcohol consumption compared with men so if the effect is via alcohol consumption, then associations between genetic variants and outcome should be null in women (assuming they consume very low to zero alcohol).^36–38^

## Educational-attainment PGS and childhood phenotypes in UK Biobank

We sought to use a population invariant for educational attainment (as measured by years in schooling) and an educational-attainment PGS to investigate the ‘threshold-exclusive model’. We used self-reported childhood phenotype data from adults in UK Biobank; relative body size at age 10 years, wearing glasses by age 15 years and smoking initiation by age 15 years. The minimum school leaving age during this time period was 15 years (or 16 years from 1972 onwards) so associations between the educational-attainment PGS and these childhood phenotypes cannot relate to effects of additional educational attainment, but could relate to effects of liability to educational attainment.

Using a sample of 337 006 individuals of recent European ancestry, we found strong evidence that a 1 standard deviation (SD) higher educational-attainment PGS (173 independent variants with *P* < 1 x 10^–^^5^ from an independent sample) is associated with higher odds of wearing glasses [odds ratio (OR) 1.05; 95% confidence interval (CI) 1.04, 1.06] and lower odds of smoking initiation (OR 0.88; 95% CI 0.87, 0.89), both at age 15 years. Contrastingly, we did not find strong evidence for an association between the educational-attainment PGS and body size at age 10 years (per SD increase in PGS: Beta = 0.000; 95% CI –0.002, 0.003), although the measure was ordinal rather than continuous, limiting power (Table  1). These results suggest that liability to educational attainment increases the wearing of glasses and reduces smoking initiation independently of years of educational attainment.

**Table 1:** Educational-attainment PGS and pre-adulthood BMI, smoking and glasses use

Outcome		Change per 1 SD increase in educational-attainment PGS (95% CI)
Body size at age 10 years (0, 1, 2)^a^	Beta (95% CI)	0.000 (–0.002, 0.003)
Wears glasses at age 15 years (yes/no)	OR (95% CI)	1.05 (1.04, 1.06)
Smoking at age 15 years (yes/no)	OR (95% CI)	0.88 (0.87, 0.89)

a Body size at age 10 years compared with peers; 0 = thinner, 1 = average, 2 = plump.

BMI, body mass index; PGS, polygenic score.

To investigate whether family-level factors (e.g. effects of parental educational attainment on offspring phenotypes^39^) or demography (assortative mating, population stratification) were driving these associations, we repeated analyses for glasses wearing and smoking phenotypes using a within-sibship model (robust against these potential biases^40^^,^^41^) in UK Biobank. Using a sample of 41 497 siblings from 19 588 sibships, we found consistent evidence that the educational-attainment PGS was associated with increased glasses wearing (per SD increase in PGS; OR 1.05; 95% CI 1.01, 1.10) and reduced smoking initiation (per SD increase in PGS; OR 0.89; 95% CI 0.84, 0.94) at age 15 years. These results are further indicative of the effects of liability to education independently of additional educational attainment.

Information on UK Biobank and the PGS analyses are contained in the Supplementary Methods (available as Supplementary data at *IJE* online).

## Raising of the school age and liability to educational attainment

An alternative, and widely used, source of evidence about the effects of educational attainment are policy reforms that affected the amount of schooling that people received—such as the raising of the school leaving age (ROSLA). In 1972, the minimum school leaving age was increased by 1 year from 15 to 16 years old. This meant that individuals born before September 1957 could leave at age 15 years, whereas those born in September 1957 or afterwards had to stay in school until at least age 16 years. ROSLA has been previously used to estimate the effects of an additional year in full-time education on health outcomes in UK Biobank.^27^

Here, we use the schooling reform to investigate whether individuals who would have chosen to stay in full-time education to 16 years even before the reform have better outcomes than individuals who would prefer to leave; this is a measure of liability to educational attainment. To distinguish this analysis from the ROSLA analyses described above, which investigate the effects of additional educational attainment, we refer to this analysis as ROSLA-L. We selected participants who reported leaving school at age 16 years in the year before the reform (born in September 1956—August 1957) and the year after the reform (born in September 1957—August 1958). This sample consists of individuals who were not affected by the reform (pre-reform cohort), who chose to remain in school until age 16 years and who were affected by the reform and may have been forced to stay until age 16 years (post-reform cohort). The post-reform cohort will include individuals who would have preferred to leave full-time education at age 15 years given the choice. We estimated the difference in outcomes between the pre- and post-reform cohorts. Assuming no time effects, differences in outcomes between the pre- and post-reform cohorts are likely to reflect group-level differences between individuals choosing to stay in full-time education until 16 years and individuals who would have preferred to leave at 15 years.

In UK Biobank, there were 2592 participants in who left full-time education at age 16 years in the pre-reform cohort and 4064 who left full-time education at 16 years in the post-reform cohort from England and Wales. Individuals in the post-reform cohort had a lower average educational-attainment PGS (–0.06 SD; 95% CI –0.11, –0.01) than the pre-reform cohort, indicative of differences in liability to educational attainment as hypothesized. We found strong evidence that the post-reform cohort had higher pack-years of smoking, a lower proportion with household income of >£18 000 and higher deprivation than the pre-reform cohort. Contrastingly, there was little evidence for group-level differences in BMI or systolic blood pressure (SBP), but the conclusions are limited by modest sample sizes (Table  2).

**Table 2:** Adulthood phenotypic differences between the pre- and post-reform cohorts

**Phenotype** **(study baseline)**	Pre-reform (*N* = 2592)	Post-reform (*N* = 4064)	Heterogeneity
	Left school at age 16 years the year before reform	Left school at age 16 years the year after reform	*P*-value
BMI: mean (SD)	27.9 (5.0)	28.0 (5.0)	0.40
Smoking pack-years: mean (SD)	21.8 (15.3)	23.7 (17.1)	0.0095
SBP: mean (SD)	136.6 (18.0)	136.2 (18.0)	0.35
Townsend deprivation index: mean (SD)^a^	–1.33 (3.0)	–0.93 (3.1)	2.6 × 10^–7^
Household income (% with >£18 000)	83.2%	80.5%	0.0080
Glasses wearing^b^ (yes/no)	90.2%	88.0%	0.0051

a A greater score implies a greater degree of deprivation.

b Includes contact lenses.

BMI, body mass index; SBP, systolic blood pressure.

The ROSLA-L results suggest that individuals with higher liability to educational attainment smoke less, have lower deprivation, have higher income and are more likely to wear glasses independently of measured educational attainment (as both cohorts left school at age 16 years). However, we cannot determine whether these associations relate to direct effects of liability to educational attainment or reflect effects of one component of liability (e.g. effects of parental educational attainment) but not all of the components (which would be effects of liability).

Information on the ROSLA-L analysis is contained in the Supplementary Methods (available as Supplementary data at IJE online).

## Discussion

Here, we discuss how to interpret Mendelian-randomization studies with non-continuous (binary or ordinal categorical) exposures, extending previous literature on binary exposures.^24^^,^^25^^,^^27^ Phenotypic variation in binary disease outcomes is often modelled using liability^13^—an underlying latent continuous trait reflecting genetic, environmental and stochastic components—and we describe how the liability model can also be applied to ordinal categorical traits. Interpretation of the effects of non-continuous exposures from Mendelian-randomization analyses is nuanced because genetic instruments for categorical exposures capture liability to the categorical exposure rather than purely variation in the categorical exposure itself. Interpreting effect estimates in terms of the categorical exposure requires making assumptions about how genetic instruments influence the outcome, either by their effect on the exposure category (‘threshold-exclusive’ model), their effect on the underlying liability (‘liability-exclusive’ model) or some combination of both (‘combined’ model).

We discussed how this assumption can potentially be evaluated by determining whether genetic instruments are associated with the outcome in subgroups in which the exposure category is invariant, such as selecting a subgroup of the population who cannot have the categorical exposure, e.g. testicular cancer in women. We recommend only stratifying on factors that cannot be influenced by the exposure and outcome (e.g. sex or age) because stratification on other factors could induce collider bias, complicating interpretation. Similar negative control approaches have been used previously in Mendelian-randomization analyses as sensitivity analyses to test whether genetic instruments are associated with the outcome when the exposure is invariant. For example, sex-stratified analyses have been used in the context of alcohol consumption in East Asian populations exploiting alcohol-behaviour differences between men and women.^36–38^ Previous studies have also looked at associations between PGS for adulthood diseases and cigarette smoking and outcomes in cohorts of children.^30^^,^^42^^,^^43^ Here, associations are likely to relate to effects of disease liability because children are unlikely to have experienced disease events or started smoking (if under a certain age).

For educational attainment, one could similarly perform analysis using phenotypes from individuals under the minimum school leaving age, as effects of the genetic instruments in this population cannot be via additional schooling. For example, previous studies have showed that educational-attainment PGS are associated with childhood school performance suggestive of liability-exclusive effects.^44–46^ Using this approach, we found evidence that educational-attainment PGS are associated with glasses use and smoking initiation by age 15 years as recalled by adult UK Biobank participants. These associations were replicated in within-sibship models suggesting that the associations were not driven by indirect genetic effects, assortative mating or population stratification.^41^^,^^47^^,^^48^ One limitation of this analysis is that the childhood phenotypes were recalled by adult study participants decades later so are susceptible to measurement error. An orthogonal approach, ROSLA-L, using a policy change also provided evidence that individuals with higher liability to education have better later-life outcomes independently of schooling. The PGS and ROSLA-L results indicate that liability to educational attainment likely affects health and social outcomes independently of educational thresholds. For example, individuals who are more likely to enrol in additional education may smoke less independently of educational attainment.

This has implications for Mendelian-randomization analyses of the effects of educational attainment; genetic variants are proxying for liability to educational attainment so causal estimates are unlikely to reflect the pure effects of an additional year of education. Indeed, Mendelian-randomization estimates scaled in terms of years in full-time education should not be interpreted as indicating purely threshold-exclusive effects of remaining in school for an additional year. Mendelian randomization has been previously used to demonstrate that measured educational attainment influences myopia and smoking behaviour.^49^^,^^50^ The associations we report here between the educational-attainment PGS and related outcomes in 15-year-olds suggest that the reported effects of additional educational attainment on these outcomes are unlikely to be purely due to whether a participant remained in school to age 15, 16, 18 or 21 years.

In general, genetic instruments are unlikely to only affect an outcome via changes in exposure category but this may be the case when an intervention relates to a specific threshold. For example, being prescribed statins may relate to cholesterol levels reaching a certain threshold or being born with a birth defect (e.g. an orofacial cleft) could result in disrupted schooling because of surgical interventions.^51^ Mendelian-randomization analyses with ordinal categorical exposures can be interpreted in terms of the effects of liability that act via and independently of the categorical exposure. As with conventional Mendelian-randomization analyses, interpretation is also sensitive to heterogeneity of the estimate effect of the exposure across the genetic instruments and directional pleiotropy, with a liability model assuming that all genetic instruments have consistent effects on the outcome. For example, our findings suggest that Mendelian-randomization analyses of educational attainment should be interpreted as the effects of liability to educational attainment that are likely to be mediated by a combination of measured educational attainment and other independent factors. We note that conventional Mendelian randomization, which divides by the genetic variant–categorical exposure association, may provide biased estimates of the total effect of liability because there are pleiotropic pathways from the genetic variant to the outcome within levels of the categorical exposure. With some additional steps, the total effect of liability can be estimated assuming a liability-threshold model for the genetic variant–categorical exposure relationship.^52^ Identifying this point estimate requires additional assumptions about the homogeneity of the genetic instruments and the relationship between the exposure and the outcome.^53^ In principle, future work could combine prospective data and dated events with Mendelian randomization to disentangle threshold and liability-exclusive effects as such an investigation is limited with existing data sets.

In comparison to Mendelian randomization, ROSLA analyses^27^^,^^54–56^ will identify the effects of continuous educational attainment (i.e. days in the classroom) and also the effects of getting any qualifications (the threshold). ROSLA estimates using the entire sample will not capture the effects of individual liability to educational attainment, as population-level average liability to educational attainment is unlikely to have changed before and after the reform. However, the average liability to educational attainment of those who chose to remain in school to age 16 years before the reform is likely to be higher than that of those who remain in full-time education to age 16 years after the reform, as the latter includes individuals who would otherwise have left at age 15 years.

To conclude, we have demonstrated that interpreting Mendelian randomization with non-continuous categorical exposures requires assumptions about the underlying causal model. We described how this assumption could be evaluated using subsets of the population invariant for the exposure. However, the practical uses of this test are limited because stratifying can induce collider bias unless the stratifying factor cannot be influenced by the exposure or outcome. Evidence from genetic and school-reform analyses suggested that liability to educational attainment affects social and health outcomes in UK Biobank independently of categorical measures of education attainment. Mendelian-randomization studies with categorical exposures such as disease status or educational attainment should be interpreted in terms of liability, which may act via pathways through the categorical exposure and via independent pathways.

## Supplementary data

Supplementary Methods are available at *IJE* online.

## Ethics approval

This research has been conducted using the UK Biobank resource under Application Number 8786. UK Biobank has ethical approval from the North West Multi-centre Research Ethics Committee (MREC).

## Funding

This research has been conducted using the UK Biobank resource under Application Number 8786. The Medical Research Council (MRC) and the University of Bristol support the MRC Integrative Epidemiology Unit [MC_UU_00011/1]. N.M.D. is supported by an Economics and Social Research Council (ESRC) Future Research Leaders grant [ES/N000757/1] and the Norwegian Research Council Grant number 295989. No funding body has influenced data collection, analysis or its interpretation.

## Data availability

This study used individual participant data from the UK Biobank with field IDs listed in the ‘Methods’ section. For details on accessing UK Biobank data, please contact access@ukbiobank.ac.uk. All summary data are contained in the article or available upon reasonable request.

## Supplementary Material

dyab208_Supplementary_Data
