# Supplementary material for: Interpreting Mendelian-randomization estimates of the effects of categorical exposures such as disease status and educational attainment
Source: Int J Epidemiol. 2021 Sep 27;51(3):948–57. doi: 10.1093/ije/dyab208 (PMC9189950; doi:10.1093/ije/dyab208)
Supplement: dyab208_Supplementary_Data [file dyab208_supplementary_data.docx]

# Interpreting Mendelian randomization estimates of the effects of categorical exposures such as disease status and educational attainment

# Supplementary Methods

UK Biobank

We used data from UK Biobank, a large prospective cohort study of 503,325 individuals, aged between 38-73 years at baseline, who were recruited between 2006 and 2010 from across the United Kingdom. The vast majority of study participants were genotyped, completed questionnaire data at baseline and have linked records with secondary care data and other health registries. The cohort has been described in detail in previous publications, including information on genotyping ^1, 2^.

For the PGS analyses, we used childhood phenotypes recalled by UK Biobank study participants in questionnaire data. Study participants were asked to describe their comparative body size at age 10 in relation to their peers (field ID 1687) as either “Thinner”, “Plumper” or “About Average”. We used this variable to construct an ordered categorical childhood BMI measure (Thinner 0, About Average 1, Plumper 2). Study participants were asked if they currently wear glasses (or contact lenses) (field ID: 2207) and if answering yes were asked the age that they started wearing them (field ID: 2217). We used these variables to create a binary variable reflecting glasses use at age 15. Individuals who reported not currently wearing glasses or reported starting use after the age of 15 were set to 0. Individuals who started wearing glasses at 15 or younger were set to 1. Study participants were asked their smoking status (field ID: 20116) and if they reported previous or current smoking were asked their age of smoking initiation (field IDs: 2867, 3436). We used these variables to create a binary variable reflecting smoking initiation at age 15. Individuals who reported being never smokers or who reported initiation after the age of 15 were set to 0. Individuals reporting smoking initiation at 15 or younger were set to 1.

For the ROSLA-L analysis, we used country of birth (within the UK) (field ID: 1647) and a measure of educational attainment based on self-reported age when leaving full-time education (field ID: 845) to define individuals who left school at aged 16 in England or Wales. A measure of lifetime smoking was generated using “pack years of smoking” (field ID: 20162), available for current or former smokers, with lifetime smoking set to zero for self-report never smokers (field ID: 20116). BMI was measured using height and weight measured during the initial assessment centre visit (field ID: 21001). Systolic blood pressure was measured using an Omron device (field ID: 4080) at study baseline. Townsend deprivation index (TDI) (field ID: 189) was measured at recruitment and is based on the participant’s postcode and regional measures of deprivation. A higher TDI score suggests higher levels of deprivation. Information on income (field ID: 738) was obtained at baseline using the touchscreen questionnaire. Participants were asked to report the category relating to their average total household before tax in British pounds (“Less than 18,000”, “18,000 to 30,999”, “31,000 to 51,999”, “52,000 to 100,000”, “Greater than 100,000”). For the purposes of this study, we created a binary variable (0 if individuals reported household income less than 18,000 and 1 if individuals reported any of the categories with an income greater than 18,000). Any individuals reporting “do not know” or “prefer not to answer” were set to missing. Glasses use was defined using the baseline questionnaire variable on whether individuals wear glasses (or contact lenses) (field ID: 2207).

We used summary data from a previous Genome-wide association study (GWAS) independent of UK Biobank to identify genetic variants putatively associated with educational attainment (P < 1×10^-5^) ^3^. Sets of independent variants were generated by LD clumping (R^2^ < 0.001, 10000 kb distance for clumps) the summary data in PLINK v1.9 ^4^. Weighted genetic risk scores were then constructed in UK Biobank study participants using GWAS summary data beta coefficients in PLINK v1.9 ^4^.

Genetic analyses

Starting with the full sample of UK Biobank participants with genotype data, we restricted to individuals of “White British ancestry” as self-reported and verified by principal components analysis. We then removed closely related individuals identified using an in-house algorithm which used the UK Biobank provided listed of related pairs to preferentially remove individuals related to the greatest number of other individuals until no related pairs remained ^5^. The final sample included 337,006 individuals. More information on the internal quality control of UK Biobank data is contained in a previous publication ^5^.

We then calculated the association between the educational attainment PGS and the childhood phenotypes described above (BMI, glasses use, smoking initiation) using regression (logistic/linear) models adjusting for sex, birth year and the first 10 principal components.

As a sensitivity analysis, we also applied within-sibship models using a sample of 41,497 siblings from 19,588 sibships from UK Biobank. Siblings were identified using UK Biobank provided measures of IBS (kinship) and IBS0 (proportion of null loci) ^6^. Within-sibship models were as above but also included the sibship mean PGS (the mean PGS amongst all siblings in a sibship) as a covariate to account for parental genotypes ^6, 7^. To account for collinearity between siblings, standard errors were clustered by sibship.

Raising of the school leaving age analysis

For the purposes of the ROSLA analyses, we restricted the sample to individuals self-reporting that they were born in England or Wales. We then defined the pre-reform cohort as individuals born between September 1^st^, 1956 and August 30^th^, 1957 who self-reported leaving school at the age of 16. The post-reform cohort was similarly defined as individuals born between September 1^st^, 1957 and August 30^th^, 1958 self-reporting leaving school at 16. In the combined sample, we then evaluated differences between the groups using a linear (Education PGS, BMI, SBP, smoking, TDI) or logistic (income > £18,000, glasses use yes or no) model with a “reform” covariate (0 for pre-reform and 1 for post-reform). Heterogeneity p-values were defined as the regression p-value of the reform covariate.

Caveats with the ROSLA approach in UK Biobank, such as the correlation between birth month and self-report school leaving age, have been discussed previously ^8^.

# Code availability

Statistical code for regression models is available on GitHub (https://github.com/LaurenceHowe/LiabilityScripts/blob/main/regression-models.R). Other queries can be addressed to the corresponding author (Laurence.Howe@bristol.ac.uk).

# References

1. Sudlow C, Gallacher J, Allen N, et al. UK Biobank: an open access resource for identifying the causes of a wide range of complex diseases of middle and old age. *PLoS Medicine* 2015; **12**: e1001779.

2. Bycroft C, Freeman C, Petkova D, et al. The UK Biobank resource with deep phenotyping and genomic data. *Nature* 2018; **562**: 203.

3. Okbay A, Beauchamp JP, Fontana MA, et al. Genome-wide association study identifies 74 loci associated with educational attainment. *Nature* 2016; **533**: 539.

4. Purcell S, Neale B, Todd-Brown K, et al. PLINK: a tool set for whole-genome association and population-based linkage analyses. *The American Journal of Human Genetics* 2007; **81**: 559-75.

5. Mitchell RE, Hemani G, Dudding T, Paternoster L. *UK Biobank Genetic Data: MRC-IEU Quality Control, version 1, 13/11/2017*. 2017.

6. Brumpton B, Sanderson E, Hartwig FP, et al. Within-family studies for Mendelian randomization: avoiding dynastic, assortative mating, and population stratification biases. *Nature Communications* 2020: 602516.

7. Davies NM, Howe LJ, Brumpton B, Havdahl A, Evans DM, Davey Smith G. Within family Mendelian randomization studies. *Hum Mol Genet* 2019; **28**: R170-r9.

8. Davies NM, Dickson M, Davey Smith G, van den Berg GJ, Windmeijer F. The Causal Effects of Education on Health Outcomes in the UK Biobank. *Nat Hum Behav* 2018; **2**: 117-25.
